# A modeling study of budding yeast colony formation and its relationship to budding pattern and aging

**DOI:** 10.1371/journal.pcbi.1005843

**Published:** 2017-11-09

**Authors:** Yanli Wang, Wing-Cheong Lo, Ching-Shan Chou

**Affiliations:** 1 Department of Mathematics, The Ohio State University, Columbus, Ohio, United States of America; 2 Department of Mathematics, City University of Hong Kong, Hong Kong, China; University of California Irvine, UNITED STATES

## Abstract

Budding yeast, which undergoes polarized growth during budding and mating, has been a useful model system to study cell polarization. Bud sites are selected differently in haploid and diploid yeast cells: haploid cells bud in an axial manner, while diploid cells bud in a bipolar manner. While previous studies have been focused on the molecular details of the bud site selection and polarity establishment, not much is known about how different budding patterns give rise to different functions at the population level. In this paper, we develop a two-dimensional agent-based model to study budding yeast colonies with cell-type specific biological processes, such as budding, mating, mating type switch, consumption of nutrients, and cell death. The model demonstrates that the axial budding pattern enhances mating probability at an early stage and the bipolar budding pattern improves colony development under nutrient limitation. Our results suggest that the frequency of mating type switch might control the trade-off between diploidization and inbreeding. The effect of cellular aging is also studied through our model. Based on the simulations, colonies initiated by an aged haploid cell show declined mating probability at an early stage and recover as the rejuvenated offsprings become the majority. Colonies initiated with aged diploid cells do not show disadvantage in colony expansion possibly due to the fact that young cells contribute the most to colony expansion.

## Author summary

Budding yeast is a model organism in understanding fundamental aspects of eukaryotic cells, such as cell polarization and cell aging. Previously, extensive research has focused on the molecular mechanisms of biological processes in yeast, but many questions regarding yeast budding remain unsolved. For example, how do different budding patterns affect yeast colony growth? How does declined spatial order due to aging impact the colony at the population level? To address these questions, we developed a computational agent-based model, which incorporates key biological processes, the effect of aging, as well as cell-environment interaction. We performed and analyzed a large number of simulations for a variety of situations, and obtained insightful results. We found that axial budding pattern enhances the percentage of diploid cells at early stage and bipolar budding pattern improves colony development under nutrient limitation; the frequency of mating type switch might control the trade-off between diploidization and inbreeding; aging affects the percentage of diploid cells in colonies initiated by a single haploid cell, but does not have much influence in the expansion of colonies initiated by diploid cells. The framework of the model can be extended to study other important systems, such as tissue with stem cell lineage.

## Introduction

**Fig 1:**
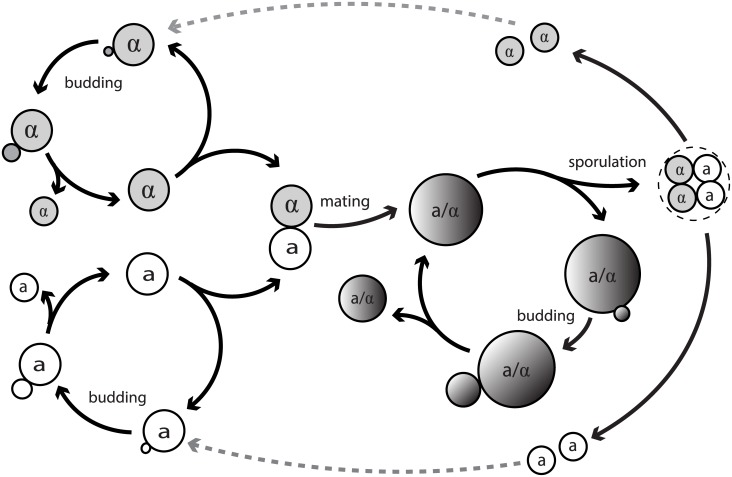
The life cycle of budding yeast.

Budding yeast *Saccharomyces cerevisiae* has been an ideal model system to study many biological processes crucial to the development of uni-cellular or multi-cellular organisms, such as cell polarization, cytokinesis and cell aging. It became a favorable model system because of its experimental tractability and the existing extensive studies over the decades. Yeast cells exist in haploid and diploid forms and they form colonies via sexual or asexual reproduction depending on the environmental cues [1]. Both haploid and diploid yeast cells can reproduce asexually by budding, in which a small bud emerges from the mother cell, enlarges until reaching a certain size, and then separates from the mother cell. The haploid cells have two mating types **a** and *α*, and they mate with their mating partners of the opposite mating type to form a diploid cell of type **a**/*α*. Under extreme conditions such as stress or starvation, diploid cells can undergo sporulation, by entering meiosis and producing four haploid spores [1, 2]. The life cycle of budding yeast is illustrated in Fig 1.

Yeast budding is an important process to understand cell polarization and symmetry breaking. Studies using both experimental or modeling approaches have been extensively conducted on yeast budding [2–5] During budding, a new daughter cell emerges from a mother cell through polarized cell growth [2]. Haploid cells bud in an axial manner in which both mother and daughter cells have their bud sites adjacent to their previous division sites; diploid cells bud in a bipolar manner in which mother cells have a new bud site either adjacent or opposite to the previous division site, whereas daughter cells mostly choose a new bud site opposite to their birth site [1, 2, 6]. This budding event involves a key polarized protein, Cdc42 GTPase, which is highly conserved from yeast to human and plays a central role in polarity establishment [7–9]. Cdc42 localizes and interacts with other players in the pathway that ultimately lead to polarized growth and the emergence of a bud [2, 4]. This polarization is oriented by spatial cues that are distinct in each cell type [6]: proteins such as Bud3 and Bud4 are thought to function as a transient spatial cue in the axial budding pattern [10, 11], while Bud8 and Bud9 are the spatial cues in the bipolar budding pattern [12]. Previous efforts have been made to understand bud emergence at the molecular and mechanistic level; however, not much is known about why the haploid and diploid cells bud in different patterns. A long-standing speculation is that different budding patterns give rise to different biological functions specific to each cell type [3, 4, 13]: the axial budding pattern may facilitate mating by generating a tighter cluster of cells with opposite mating types, and the bipolar budding pattern may maximize the expansion of the colony, allowing a wider nutrient search in new territory.

An interesting feature of haploid budding yeasts is their ability to switch the mating type. Homothallic haploid yeast strains are able to switch between two mating types during mitotic growth [1, 14] and generate a colony that is a mixed population of both haploid and diploid cells. Mating type switch has an advantage of allowing haploid cells to change their mating type in daughter cells to generate a compatible mating partner, but it may come at a cost of forming diploid cells between closely related cells (mother-daughter or siblings), resulting in inbreeding which reduces genetic variation and fitness of offsprings [1]. How the cells balance these benefits and costs from mating type switch is yet unclear and could be related to the frequency of mating type switch [1, 14–16].

An unavoidable factor affecting all the processes discussed above, as well as almost all the other biological functions, is aging. Budding yeast renders itself as a useful tool to study the evolutionary conserved aspects of eukaryotic aging [17]. Individual yeast cells divide limited times before they die, and the number of cell divisions is defined as their replicative age [18]. It is known that certain cellular functions or processes are associated with replicative age, for example, the mortality rate, cell cycle length, cell size and the sensitivity to environment (such as response to mating pheromone and nutrients) [18]. It was also observed that the cellular spatial order declines with replicative age, and interestingly, by tracking individual yeast cells, experiments showed that the probability of normal budding decreases with age [19, 20]. While it is still elusive whether the change of budding pattern is the cause or consequence of aging, a natural but unanswered question is how this loss of correct orientation in old cells impacts the colony at the population level.

Mathematical modeling has served as a useful tool to successfully address many important questions regarding cell polarization. Similar to the previous experimental works on yeast, most modeling works for budding yeast are on the molecular level to understand the pathways and mechanisms in cell polarization [5, 21, 22]. Modeling works that study yeast from the population point of view is very limited. In [23], an agent-based model was proposed to study the effects of different budding patterns and growth inhibition (induced by crowding effect) on colony morphology at the single-cell level. Their simulations demonstrated that growth inhibition and polar budding pattern are the most significant factors driving colony expansion. In [24, 25], agent-based models were proposed to simulate yeast colony growth, which includes a size-controlled module to govern cell proliferation and a cell-cell interaction module to arrange spatial positions of cells; the authors discussed the influence of cell-cell cohesion force and budding patterns on the colony shape and size [24], and they studied a variety of diameter growth time and reproduction time to better match the exponential growth in experiments [25]. However, the studies in [23–25] did not consider the intrinsic difference between budding patterns of haploid and diploid cells, nor did they discuss how budding patterns and cell types affect the growth of colonies. In addition, the existing models did not include the interaction between cells and their living environment.

In this paper, we present a novel and more comprehensive agent-based type model to study how the budding patterns in yeast cells affect colony growth. Our model incorporates many important biological processes in yeast cells, colony spatial arrangement through cell-cell mechanical iterations, and cell-environment interactions. To be more specific, the key biological processes include budding, mating, mating type switch, changes in cell cycle length and cell size due to aging, and cell death; cell-cell mechanical interaction is modeled through a contractive component due to cell adhesion and a repulsive component due to elastic compression [26–29]; a nutrient field is introduced and the nutrient is consumed by cells while growth inhibition is induced if the nutrient level is too low. Our major findings include that (1) mating type switch frequency controls the trade-off between diploidization and inbreeding; (2) axial budding pattern in haploid yeast cells facilitates mating at an early stage of colony expansion; (3) bipolar budding is necessary for a branched colony under limited nutrient; (4) mating efficiency is lower in aged colonies but colony expansion does not depend on the overall age of the colonies. It is worth remarking that our modeling framework is not restricted to budding yeast and could be applied to study other systems, such as fungi, bacteria and stem cells. The paper is structured as follows. A detailed description of the model is given in Models Section. In Results Section we present and analyze the results. Conclusions and discussions are given in Discussion Section. Supporting figures and texts can be found in Supporting Information.

## Models

In nature, yeast cells bud and mate in a three-dimensional space and change their shapes during these processes. In our model, the cross sections of yeast cells on a two-dimensional domain are considered and their shapes remain spherical before and after budding and mating. This simplification would not significantly impact our conclusions since the focus of our study will be on the total population of yeast colonies and the overall spatial distribution.

**Fig 2:**
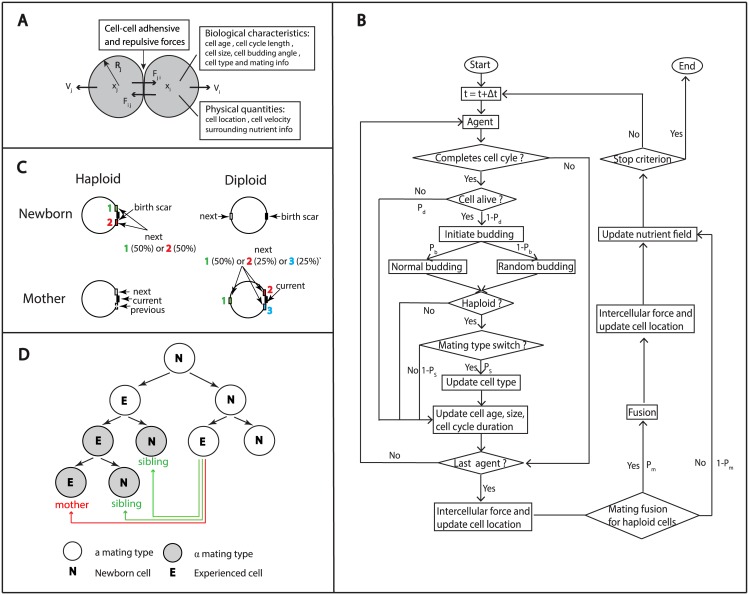
(A) A schematic of the agent-based model, with the key biological and physical quantities. (B) Overview of the processes within a single cell cycle. *P*_*d*_ and *P*_*b*_ are the probabilities of cell death and normal budding (axial for haploid cells and bipolar for diploid cells), respectively. *P*_*s*_ and *P*_*m*_ are the frequencies of mating type switch and successful matings for haploid cells. The simulation stops when the maximal time or the maximal population is attained. (C) Normal budding patterns for haploid and diploid cells. Haploid cells bud in an axial manner: both mother and daughter cells have bud sites adjacent to the previous division site. Diploid cells bud in a bipolar budding pattern: mother cells have a new bud site adjacent to their daughters or on the opposite end of the cell, whereas daughter cells mostly choose a new bud site on the opposite end of the cell. (D) Mating type switch follows certain rules: (1) only experienced cells can switch mating type; (2) mating type switch occurs during the late G1 phase and the switched cells come in pairs; (3) mating type switch occurs at a high frequency. Inbreeding is defined as mating between mother and daughter cells or among siblings.

Our model considers both haploid cells, which are of either **a** or *α* mating type, and diploid cells, which are of **a**/*α* type. Each cell is viewed as a single agent and carries its own biological and physical information (summarized in Fig 2A). As time progresses with discrete time steps, the information will be updated with certain rules which will be further explained in details in the remainder of this section. During each time step, cells may experience budding, cell death, mating (haploid cells) or mating type switch (haploid cells); cell size and cell cycle length may change depending on the age of cells or other factors, and cells’ location may be rearranged due to budding or mating when the number of cells changes. In the extracellular space, there is a nutrient field which is initially set to be uniform and is updated at each time step due to the consumption by cells. In the meanwhile, cell cycle length may be prolonged by nutrient deficiency. The agent-based algorithm is summarized in a flow chart in Fig 2B and the parameters used in the simulations are shown in Table 1.

**Table 1:** Parameters used in simulations and their references.

Parameter	Value	Reference
*k*_1_	0.006 (haploid) 0.004 (diploid)	Estimated from [20]
*k*_2_	0.024	Estimated from [19]
*a*_*d*_	30	Estimated from [30, 31]
*a*_*h*_	25	Estimated from [30, 31]
*g*_0_	101.25	Estimated from [30, 31]
*g*_11_	2/3	Estimated from [30, 31]
*g*_12_	2/3	Estimated from [30, 31]
*k*_3_	80	This paper
*D*	0	This paper
*c*_*r*_	0.6	This paper
*c*_*d*_	2	This paper
*U*_0_	[1, 2]	This paper
$c_{u}^{1}$	3	This paper
$c_{u}^{2}$	1	This paper
$c_{u}^{3}$	2	This paper
$c_{u}^{4}$	50	This paper
*d*_*nur*_	2	This paper
*k*_4_	45	This paper
*k*_*r*_	1	This paper
*k*_*a*_	0.4	This paper
*α*	500	This paper
*c*_1_	50	This paper
*c*_2_	50	This paper
*c*_3_	100	This paper
*c*_4_	100	This paper
*c*_5_	100	This paper
*c*_6_	120	This paper
Δ*t*	1.8	This paper
Δ*x*, Δ*y*	0.7	This paper

### Cell death

The lifespans of yeast cells can be measured by either their replicative potential (replicative lifespan) or the maximal survival time of a non-dividing cells (chronological lifespan) [17, 18]. In the literature both lifespans are used to study different aspects of aging: the replicative lifespan is associated with the total number of cell division, and the chronological lifespan is related to the physical time. Only replicative lifespan is considered in this paper because we are mainly interested in the budding event and budding patterns.

It is known that during budding, yeast cells undergo asymmetric division, in which mothers give rise to daughter cells with full lifespan capacity [18]. Therefore, in our model, upon cell division the age of the mother cell is increased by one, while the initial age of daughter cell is set to be zero. In experiments, the replicative lifespan is measured by counting the number of total bud scars [19, 20], and the average replicative age is approximately 30–50 cell divisions [20].

The death probability, denoted by *P*_*d*_(*a*), represents the probability that a cell with age *a* dies before reaching age *a* + 1. Although this important quantity is not directly observable, its companion, the survival fraction *F*_*s*_(*a*) of the population, can be measured in experiments. Since the survival fraction *F*_*s*_(*a*) can be viewed as the probability that cells survive up to an age greater than *a*, *F*_*s*_(*a*) and *P*_*d*_(*a*) are related by the following formula

$$F_{s}\left(a\right)=\left(1-P_{d}\left(a\right)\right)F_{s}\left(a-1\right)\;\;\;\;\text{for}\;a\geq 1,$$

with *F*_*s*_(0) = 1 − *P*_*d*_(0). Thus

$$F_{s}\left(a\right)=\prod_{i=0}^{a}\left(1-P_{d}\left(i\right)\right),\;\;\;\text{for}\;a\geq 1.$$

As cells bud many times, their death probability becomes higher [18]. In our model the death probability *P*_*d*_(*a*) is assumed to take the following form

$$\begin{array}{c} P_{d}\left(a\right)=1-e^{-k_{1}a}, \end{array}$$ (1)

and thus the corresponding survival fraction is

$$\begin{array}{c} F_{s}\left(a\right)=e^{-k_{1}\;\sum_{i=0}^{a}\;i}=e^{-k_{1}\frac{a(a+1)}{2}}, \end{array}$$ (2)

which is a sigmoid function. This is consistent with the shape of survival curves measured in experiments, regardless of the cells’ genetic background [18].

Previously, Jazwinski and Wawryn [20] measured the survival fraction of a population of haploid yeast cells through different ages. Using the experimental data in [20], the value of *k*_1_ is estimated to be 0.006 for haploid cells. As for diploid cells, there is no available data of survival fraction to our knowledge; however, it has been reported that diploid cells are longer-lived than haploid cells [32], so *k*_1_ = 0.004 is chosen for diploid cells. These estimated survival fractions are shown in S1A Fig.

### Budding patterns

Yeast cells undergo polarized division by budding at specific sites determined by their cell types. Wild type haploid cells bud in an axial manner: mother cells form new buds adjacent to previous bud site and daughter cells bud next to their birth site. On the other hand, wild type diploid cells bud in a bipolar manner: mother cells can choose a new bud site either adjacent (proximal pole) or opposite to (distal pole) the previous bud site and daughter cells bud at the site opposite to the birth site (distal pole) [10–12]. The schematic diagram of bud sites is shown in Fig 2C.

Interestingly, as a cell ages, its budding pattern, a representation of the cellular spatial order, appears to be disrupted with a manifestation of budding randomly at a higher frequency [19, 20]. Based on single-cell observations in [19], which tracked the budding patterns throughout lifespans of cells, we assume the probability of random budding to be an increasing function of the replicative age *a*:

$$\begin{array}{c} P_{b}\left(a\right)=1-e^{-k_{2}a}, \end{array}$$ (3)

where *k*_2_ is estimated to be 0.024. The estimated probability of random budding for haploid cells is shown in S1B Fig. Due to the lack of experimental data, diploid cells are assumed to have the same random budding probability as haploid cells.

### Cell size and cell cycle length

Morphological and physiological changes were observed during the aging process of yeast [17, 18]. For example, cell size and cell cycle length were shown to gradually increase with successive divisions [18]. It was shown in [31] that the average radius of diploid cells increases from 3.5*μm* to 5.5*μm* from birth to death. Cells of the first generation are usually small and require a long cell cycle to reach a critical size to bud. To simplify the calculation, we skip the growing process when daughter cells are attached to the mother cells. We model daughter cells after they detach from the mother cells and set the initial size to be nonzero. In our model, the radius of a newborn diploid cell is set to be 3.5*μm* and increases by $\frac{1}{a_{d}}$ at each division after its cell size reaches the critical size 4.375*μm*. Thus, the radius function for diploid cells can be formulated as

$$\begin{array}{c} r_{d}\left(a\right)=\{\begin{array}{cc} 3.5 & \;\text{if}\;a=0,\\ 4.375+a/a_{d} & \;\text{if}\;a>0, \end{array} \end{array}$$ (4)

where *a*_*d*_ = 30. Since the average cell size of diploid cells is approximately 1.25 times that of the haploid cells [30], the radius of a newborn haploid cell is assumed to be 2.8*μm* and increases by $\frac{1}{a_{h}}$ at each division after its cell size reaches the critical size 3.5*μm*. Similarly, the radius function for haploid cells takes the form

$$\begin{array}{c} r_{h}\left(a\right)=\{\begin{array}{cc} 2.8 & \;\text{if}\;a=0,\\ 3.5+a/a_{h} & \;\text{if}\;a>0, \end{array} \end{array}$$ (5)

where *a*_*h*_ = 25. The comparison of changes in cell size with respect to age is shown in S1C Fig.

Cell cycle lengths of budding yeast cells have been shown to increase with age [18], with possibly the exception of newborn cells because they have a longer G1 phase before the initiation of budding. While cell cycle lengths vary from cell to cell and depend on the strain background and growth media, the model is simplified by ignoring the variations among the cells and the stochasticity due to other factors. In addition, the cell cycle lengths of haploid and diploid cells are assumed to be the same because no significant difference in average generation time has been observed in experiments [30]. Thus, for both types of cells, the cell cycle length λ(*a*) is modeled as

$$\begin{array}{c} \lambda \left(a\right)=\{\begin{array}{cc} g_{0} & \;\text{if}\;a=0,\\ \frac{g_{1}}{g_{11}+g_{12}/\left(a+1\right)} & \;\text{if}\;a>0\text{.} \end{array} \end{array}$$ (6)

Based on the experimental data in [30, 31], the parameters are chosen as follows: *g*_0_ = 101.25 mins, *g*_1_ = 90 mins and $g_{11}=g_{12}=\frac{2}{3}$. The estimated curve for cell cycle length is shown in S1D Fig.

### Mating and mating type switch

Mating is a process in which a haploid **a** cell and a haploid *α* cell come into physical contact and once successful, these two cells fuse into a diploid **a**/*α* cell. It is known that haploid cells of opposite mating types tend to mate to form a diploid cell [33]. Yeast cells select their mating partners and preferentially mate with the cell that produces the highest level of pheromone [34, 35]. However, cells become less sensitive to mating pheromone and become sterile as they grow old. Experiments showed that the frequency of successful matings dropped significantly when one mating partner was relatively old [18, 35]. Interestingly, when cells of different ages mate, the replicative age of the zygote is set to be that of the older haploid cell, indicating that age is a dominant phenotype [35].

Within a colony whose haploid cells have one dominant mating type, the chances of forming a diploid cell can be enhanced by mating type switch, a process in which haploid **a** cells and *α* cells switch their mating types. The ability to switch mating type is restricted to cells that have budded at least once [1, 14]. This process is regulated by the HO gene, which may be activated in mother haploid cells during the G1 phase [16]. Mating type switch is not a rare event: previous experiments suggested that mating type switch occurs with a high frequency, usually greater than 50% [1]. Hence even if a colony starts with one single haploid cell, both **a** and *α* cells will be present in that colony. The high frequency of mating type switch and the tendency of mating between haploid cells with opposite mating types result in the prevalence of diploid cells in a colony. On the other hand, the high frequency of mating type switch may also lead to inbreeding, which reduces genetic variation [14–16].

Based on biological observations, the following simplified rules are used during the mating process in our model (Fig 2D): (i) if two haploid cells of opposite mating types are in direct contact, the frequency of successful mating *P*_*m*_ will drop as cells age:

$$\begin{array}{c} P_{m}\left(a\right)=0.75-\frac{a}{k_{3}}, \end{array}$$ (7)

where *k*_3_ is estimated as 80; (ii) newborn cells are not allowed to switch mating types, while experienced cells have a constant mating type switch frequency *P*_*s*_; (iii) cells will preferentially choose the youngest of neighbors of the opposite type to mate; (iv) a newly formed diploid cell has a circular shape and has the same age as the older haploid cell prior to mating, and its volume is the sum of two mated cells; (v) inbreeding is defined as mating between mother and daughter cells or among siblings.

### Nutrient field

The growth of individual yeast cells and the expansion of the colony depend on nutrient supply. Decrease in nutrient concentration will slow down cell growth by prolonging cell cycle length [36, 37]. The level of nutrient also affects cell-cell adhesion and cell-media adhesion because nutrient depletion may activate certain genes to express corresponding cell-wall proteins that are essential for cell filamentous growth [26].

In our model, a nutrient field *u*, as a function of space $\vec{x}$ and time *t*, is introduced across the domain. The change of nutrient concentration is due to diffusion of nutrient and consumption by live cells. The consumption rate is assumed to be highly localized around cells and decreases exponentially with the distance from cells. The dynamics of nutrient concentration is described by

$$\begin{array}{c} \frac{\partial u}{\partial t}=D\Delta u-\;\sum_{k=1}^{N(t)}\;c_{r}e^{-\frac{|\vec{x}-{\vec{x}}_{k}|}{c_{d}}}u, \end{array}$$ (8)

where ${\vec{x}}_{k}$ denotes the coordinates of the center for the *k*-th cell, *N*(*t*) is the total number of cells at time *t*, *c*_*r*_ is the consumption rate of a single cell, and *c*_*d*_ controls the degree of local consumption. The diffusion coefficient *D* is selected depending on the growth media: larger values of *D* for more liquid media and smaller values for more solid media. The initial nutrient field is assumed to be homogeneous with a value *U*_0_.

To account for the growth inhibition induced by nutrient depletion for each individual cell, the cell cycle lengths are assumed to depend on local nutrient concentration *u*_*loc*_, and therefore *g*_0_ and *g*_1_ in Eq (6) are replaced by *g*_0_ *f*(*u*_*loc*_) and *g*_1_ *f*(*u*_*loc*_), respectively, where *f* is a decreasing function. It is reasonable to assume that *f* is 1 when nutrient is rich, decreases slowly under nutrient consumption, and drops rapidly when the nutrient supply is very limited. Therefore in our model *f* is defined by *f* = max{*f*_1_, 1}, where

$$\begin{array}{c} f_{1}\left(u_{loc}\right)=\{\begin{array}{cc} c_{u}^{1}-c_{u}^{2}u_{loc} & \;\text{if}\;u_{thd}\leq u_{loc}\leq U_{0},\\ c_{u}^{3}-c_{u}^{4}\log\left(u_{loc}\right) & \;\text{otherwise}, \end{array} \end{array}$$

where $c_{u}^{1}$, $c_{u}^{2}$, $c_{u}^{3}$, $c_{u}^{4}$ and *u*_*thd*_ are constants.

In the numerical implementation, the average local nutrient concentration for a cell centered at $\vec{x}$ with radius *r* can be approximated by

$$u_{loc}\left(\vec{x}\right)\approx \frac{1}{n}\;\sum_{m}\;u_{m}\left(1-H(\right|{\vec{x}}_{m}-\vec{x}|/r-d_{nur})),$$

where *u*_*m*_ is the nutrient concentration at a grid point ${\vec{x}}_{m}$, *d*_*nur*_ is the range that a cell can sense nutrient, *n* is the total number of *m* such that $|{\vec{x}}_{m}-\vec{x}|/r\leq d_{nur}$, and *H* denotes the Heaviside function.

As a cell grows older, its response to environment becomes less sensitive. To model this effect, the sensitivity coefficients to nutrient, $c_{u}^{2}$ and $c_{u}^{4}$, are assumed as decreasing functions of age of the following form

$$c_{u}^{i}\left(a\right)=\frac{c_{u}^{i}}{1+a/k_{4}},$$

where $c_{u}^{i}$ and *k*_4_ are constants and *i* ∈ {2, 4}. The values of these parameters are shown in Table 1.

### Cell spatial arrangement

In this paper, we used the off-lattice modeling approach, in which the positions of cell are not confined on mesh grids and the colony spatial arrangement is completely determined by budding, mating (haploid) and cell-cell interactions. This modeling approach gives more freedom and higher accuracy to model the location of new bud in different budding patterns and study how they affect colony formation, which is the primary subject in our paper.

The spatial distribution of a colony of cells depends on the response of cells to forces exerted by their neighboring cells. For example, yeast cells in physical contact can form adhesive bonds, which result in adhesive force, by certain proteins located on the surface of cell walls [26, 27]. Yeast cells can also resist the compression by other cells due to the incompressibility of their cell wall [28, 29].

Many models are proposed to model cell-cell adhesive and repulsive forces [23, 38–40]. The repulsive force is mainly designed to avoid overlap between two agents in the model. Experiments have shown that that linear elastic constitutive equation can be used to describe cell wall material of a yeast cell [28, 29]. In this paper, forces between cells are modeled by linear contractile-repulsive springs as in [23]. Consider the *i*-th cell centered at ${\vec{x}}_{i}$. The repulsive force between the *i*-th cell and the *j*-th cell centered at ${\vec{x}}_{j}$ is assumed to increase with the overlap Δ*d* = (*r*_*i*_ + *r*_*j*_) − *d*_*ij*_, where $d_{ij}=\left|{\vec{x}}_{i}-{\vec{x}}_{j}\right|$ and *r*_*i*_, *r*_*j*_ are the corresponding radii of the cells. Then this repulsive force is given by

$${\vec{F}}_{r}^{i,j}=\{\begin{array}{cc} k_{r}\Delta d\;{\hat{v}}_{i,j} & \text{if}\;d_{ij}<r_{i}+r_{j},\\ 0 & \text{otherwise,} \end{array}$$

where *k*_*r*_ is a spring stiffness constant and ${\hat{v}}_{i,j}=\frac{{\vec{x}}_{i}-{\vec{x}}_{j}}{d_{ij}}$ is the unit vector in the direction of ${\vec{x}}_{i}-{\vec{x}}_{j}$. The adhesive force is assumed to be proportional to the overlap between a cell and its neighbors, and is defined by

$${\vec{F}}_{a}^{i,j}=\{\begin{array}{cc} -k_{a}\Delta d\;{\hat{v}}_{i,j} & \text{if}\;d_{ij}<r_{i}+r_{j},\\ 0 & \text{otherwise,} \end{array}$$

where *k*_*a*_ is a spring stiffness constant.

Thus the overall force exerted on the *i*-th cell centered at $\vec{x_{i}}$ is given by

$${\vec{F}}_{i}=\sum_{j=1,j\neq i}^{N(t)}\;(F_{r}^{i,j}+F_{a}^{i,j}).$$

According to the Newton’s second law, the acceleration ${\vec{a}}_{i}$ is proportional to force ${\vec{F}}_{i}$. Assuming that initial velocity of the cell is 0, the instantaneous velocity is ${\vec{V}}_{i}={\vec{a}}_{i}\Delta t$ and

$${\vec{V}}_{i}=\alpha {\vec{F}}_{i}\Delta t,$$

where *α* is taken to be constant for simplicity. Thus the current position ${\vec{x}}_{i}^{\;n+1}$ of the *i*-th cell can be approximated by

$${\vec{x}}_{i}^{\;n+1}={\vec{x}}_{i}^{\;n}+{\vec{V}}_{i}\Delta t\;\;\text{for}\;n\geq 0,$$

where ${\vec{x}}_{i}^{\;n}$ denotes the previous position of the *i*-th cell, and the time step Δ*t* is chosen as 1.8 mins in our simulations, which is sufficiently small compared to cell cycle length.

## Results

All the simulations presented in this section were based on model assumptions in Models Section, unless otherwise stated. The simulations were based on the algorithm presented in the flow chart in Fig 2B and S1 Text.

### Mating type switch frequency controls the trade-off between diploidization and inbreeding

Mating type switch allows haploid cells to divide and change their mating type to generate a compatible mating partner. A single homothallic haploid cell will generate a colony with a mixed population, which contains both diploid **a**/*α* cells and haploid cells of **a** and *α* types. Diploidization is advantageous because diploid cells are better than haploid cells at coping with DNA damage. However, mating type switch is likely to come with a cost [15]. For example, mating type switch may cause replicative delays, and the presence of switching mechanisms increases DNA replication errors. In addition, the formation of diploid **a**/*α* cells from closely related cells (mother-daughter or siblings) results in inbreeding and reduces the genetic variation, which is the primary selective force. While many homothallic strains of yeast cells switch their mating type at a very high frequency (about 70% of the total cell divisions), it is not understood why the switch does not happen at an even higher frequency, such as 100% [1, 14, 16].

**Fig 3:**
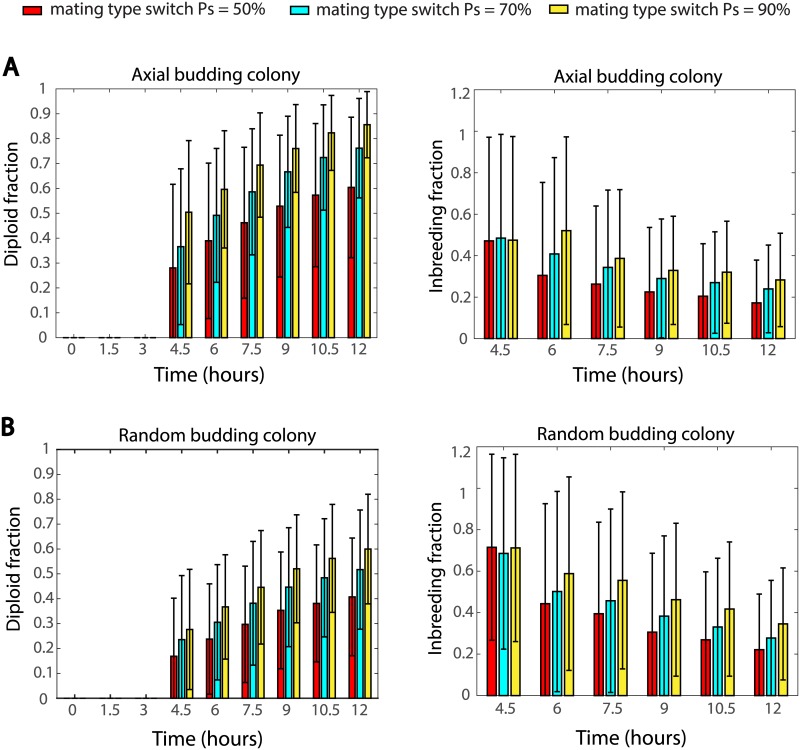
Effect of the mating type switch frequency on diploid cell percentage and inbreeding percentage in (A) axial budding colonies (B) random budding colonies. Each bar represents the average value (± standard deviation).

To study the benefit and cost of mating type switch in budding yeast colonies, we examined two corresponding indicators: the percentage of diploid cells and the percentage of inbreeding among all mated pairs. In our simulations, three frequencies, 50%, 70% and 90%, of mating type switch were tested based on a simple setting: a colony starts with a single haploid **a** cell, and this cell will bud and its offsprings are likely to switch their mating types, which eventually leads to mixed types of cells (a sample colony is shown in S2 Fig). 1000 samples were simulated for each mating type switch frequency and the statistics are summarized in Fig 3. It can be seen that, in wild type axial budding haploid cells, higher mating switch frequency leads to higher percentage of diploid cells (Fig 3A left panel) but meanwhile also leads to higher percentage of inbreeding (Fig 3A right panel). Fig 3B shows that this observation does not depend on the budding pattern of these haploid cells, and the same conclusion also holds for random budding haploid cells (new buds emerging at random positions). These results reveal the trade-off between diploidization and inbreeding controlled by the frequency of mating type switch, and may explain why the mating type switch frequency for wild type cells is approximately 70% instead of 100%. In the remainder of this paper, the mating type switch frequency is set to be 70%, unless otherwise specified.

### Axial budding pattern in haploid yeast cells facilitates mating

Different budding patterns are thought to contribute to different biological functions specific to each cell type [3, 4, 13]. Some researchers believe that axial budding pattern helps generate a tighter cluster of cells and facilitates the mating of haploid cells of opposite mating types to form diploid cells. Using our model, this hypothesis was tested by comparing colonies with axial budding haploid cells (new buds emerging adjacent to the previous bud site) and colonies with random budding haploid cells (new buds emerging at random positions). The time of first mating and the percentage of diploid cells in a colony were used as a quantitative measure to assess the mating efficiency.

**Fig 4:**
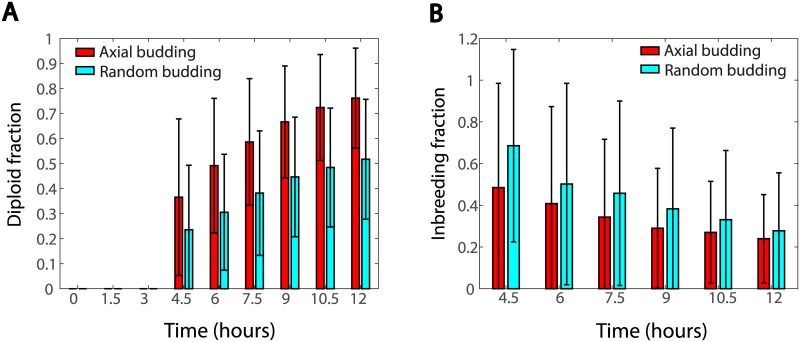
Comparisons of colonies with axial and random haploid budding patterns. (A) Percentage of diploid cells as time evolves. (B) Percentage of inbreeding calculated from the same simulations in (A).

In 1000 simulations performed for each budding pattern, we found that although the total populations for both budding patterns show exponential growth, these two budding patterns lead to significantly different mating features. On average, the first mating happens earlier in the axial budding colonies as shown in Table 2. Moreover, the axial budding colonies show significantly higher percentage of diploid cells than the random budding ones (Fig 4A). At 12 hours, over 70% of the population are diploid cells in the axial budding colonies, while the percentage for the random budding colonies is only 50%. These results support that axial budding pattern facilitates mating, especially at an early stage of colony growth.

**Table 2:** The time of first mating.

Budding pattern	mean	median	min	max	standard derivation
Axial	3.6	4.1376	3.3	10.2	1.126
Random	5.1	4.8867	3.3	10.5	1.753

Another interesting and unexpected result from the simulations is that axial budding colonies show significantly lower percentage of inbreeding during the early stage of colony development, compared to random budding colonies with the same mating type switch frequency (Fig 4B). At 4.5 hours, among all pairs of mated cells, about 65% are mother-daughter or siblings in random budding colonies, and this percentage is only around 45 for axial budding colonies. However, this difference becomes smaller as colonies grow.

### Bipolar budding is necessary for a branched colony under limited nutrient

It was hypothesized that bipolar budding is important for maximizing the spread of a colony to reach out for nutrient in new territory [3, 4, 13]. Diploid cells require the BUD8 protein for bipolar budding: *bud*8Δ mutants do not bud in a bipolar manner but instead bud adjacent to their birth scars in a pattern similar to haploid axial budding [6]. By comparing wild type cells to *bud*8Δ mutants, it was shown that bipolar budding is necessary for colony spread and agar invasion [41].

To understand the relationship between budding patterns in diploid cells and the spread of colonies, colonies with bipolar and random budding diploid cells were studied via our model. In order to assess the differences of these colonies, two indicators that measure the overall spread were introduced. The first indicator, called the colony radius and denoted by *R*, is the radius of the minimal covering circle of the colony (see S3 Fig for illustration). Larger colony radius implies a wider spread and higher efficiency in nutrient search. The second indicator, called the colony sparseness and denoted by *σ*_*D*_, is defined as the ratio between the area of the minimal covering circle and the total actual area of the colony:

$$\sigma_{D}=\frac{\pi R^{2}}{\text{area}\;\text{of}\;\text{the}\;\text{colony}},$$

where the actual area of the colony is the sum of areas of all cells. Since most of the cells do not overlap with each other in the early stage of colony formation, the sum of cell areas can be calculated according to the cell radii. Larger colony sparseness implies sparser distribution of cells inside the minimal covering circle and less competition for nutrient from neighboring cells.

**Fig 5:**
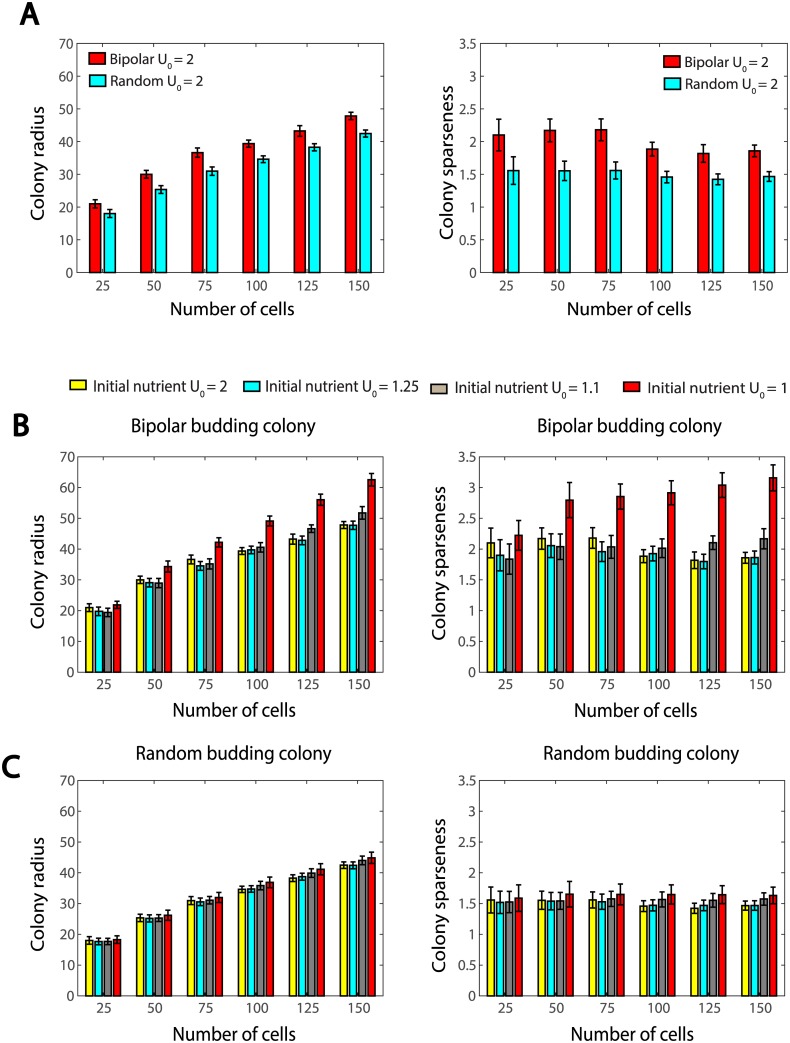
(A) Colony radius and sparseness for diploid colonies as population grows. In these simulations, we set the initial nutrient level *U*_0_ = 2. (B) Colony radius and sparseness in bipolar budding colonies as population grows. In these simulations, we set the initial nutrient level *U*_0_ = 1, 1.1, 1.25 or 2. (C) Colony radius and sparseness in random budding colonies as population grows. In these simulations, we set the initial nutrient level *U*_0_ = 1, 1.1, 1.25 or 2.

In the simulations, these two types of colonies start with four diploid cells, and will contain only diploid cells because sporulation is not considered in this paper. For each of the situations under different budding patterns and initial nutrient settings, 1000 samples were simulated and data are recorded when colonies grow to 25, 50, 75, 100, 125 and 150 cells (shown in Fig 5A).

When the initial nutrient is abundant (by setting *U*_0_ = 2), for both bipolar and random budding colonies, the colony radius increases on average as colonies grow. The bipolar budding colonies have, on average, slightly larger colony radius and sparseness than the random budding ones (Fig 5A). However, these advantages for the search of nutrient is gradually lost as colonies grow. Our simulations also agree with the modeling results in [23], which suggested that simply switching budding pattern from non-polar to polar does not necessarily lead to significant increase in colony size. Since only little advantage of the bipolar budding pattern was observed under rich nutrient condition, we tested a decreased initial nutrient level *U*_0_ = 1, which represents a poor nutrient condition.

Fig 5B shows that for bipolar budding colonies, when the initial nutrient level *U*_0_ decreases, both the colony radius and sparseness increase on average. With rich initial nutrient *U*_0_ = 2, the colony radius on average increases about 150% as the population grows from 25 cells to 150 cells, while the colony sparseness remains almost constant; on the other hand, when the initial nutrient level is lowered to *U*_0_ = 1, the increase in colony radius is over 200%, and the colony sparseness also increases more than 40%. These observations support that the bipolar budding pattern enhances colony development through better nutrient search. Another noticeable observation is that the curves of colony radius and sparseness have shifted significantly as *U*_0_ decreased from 1.1 to 1, suggesting the possible existence of certain threshold of nutrient, below which the bipolar budding is far more advantageous.

However, the situation is different for the random budding colonies. As shown in Fig 5C, nutrient limitation does not cause an active spread of the colony and there is little increase in colony radius and sparseness with various *U*_0_. Previously, Jönsson and Levchenko Jonsson2005 found that the colony area increased by 20% when neighbor inhibition varied from weak to strong, even when all divisions were non-polar. This difference between our results and theirs in [23] may be explained by the different approaches used to model the growth inhibition: in [23] the growth inhibition was modeled to be proportional to the number of neighboring cells, while in our model the growth of the cells is inhibited as their surrounding nutrient is consumed by themselves and neighboring cells. This growth inhibition due to nutrient consumption is negligible under rich nutrient condition but will be more pronounced when the overall nutrient is limited.

**Fig 6:**
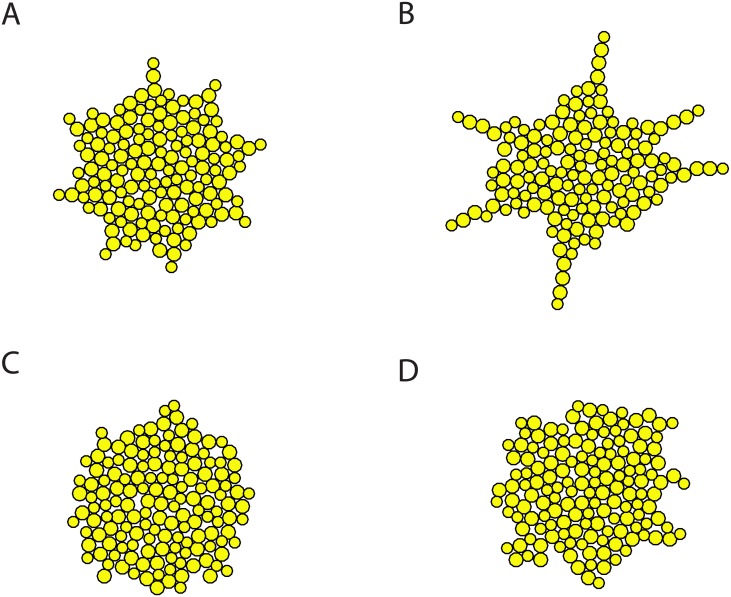
Sample simulations of colonies with 150 cells under different nutrient levels. (A) Bipolar budding under rich nutrient *U*_0_ = 2. (B) Bipolar budding under limited nutrient *U*_0_ = 1. (C) Random budding under rich nutrient *U*_0_ = 2. (D) Random budding under limited nutrient *U*_0_ = 1.

Besides the measures of colonies, we are also interested in the actual colony morphology. Under rich nutrient condition *U*_0_ = 2, colonies of both budding patterns exhibit approximately round shape except that the peripheries of the bipolar budding colonies are not as smooth and have lightly irregular extensions (Fig 6A and 6C). Under poor nutrient condition *U*_0_ = 1, bipolar budding colonies have a tight core and finger-like branches to reach out for nutrient (Fig 6B), while random budding colonies are still relatively compact with emerging small branches at the periphery (Fig 6D). More samples of colony morphology are shown in S4 Fig. Our simulations suggest that only bipolar budding cells give rise to a colony morphology with finger-like extensions under limited nutrient. Our results are consistent with the previous experiments of the lab strain S288C, which showed smooth colony structure and was specifically selected to be non-flocculent with a minimal set of nutritional requirements [41, 42].

### Mating efficiency is lower in aged colonies but colony expansion does not depend on the overall age of the colony

It is known that cellular functions decline as a cell ages. As consequences or causes, increased cell size, cell cycle length and death probability, disruption of regular budding pattern, as well as lower sensitivity to mating pheromone are manifestations of aging. Experiments have shown that daughter cells from older mothers have shorter lifespan [18]. However, granddaughters of an old mother cell show a gradual restoration to a normal lifespan, suggesting that some aging factors might be diluted to recover rejuvenation [18].

To accurately reflect the effect of the ages of mother cells as observed in biological experiments, we included a variable *a* representing age in some probabilities or parameters introduced in Models Section: (i) Daughter cells born from older mothers have higher death probability and are more likely to bud randomly. Accordingly Eqs (1) and (3) are modified to:

$$P_{d}\left(a,a_{M}\right)=1-e^{-k_{1}\left(1+a_{M}/c_{1}\right)a}\;\text{and}\;P_{b}\left(a,a_{M}\right)=1-e^{-k_{2}\left(1+a_{M}/c_{2}\right)a},$$

where *a*_*M*_ is the age of the mother cell. (ii) The initial size of a daughter cell increases with the age of its mother: for diploid daughter cells, Eq (4) is changed to 3.5(1 + *a*_*M*_/*c*_3_)*μ*m, and for haploid daughters, Eq (5) is modified as 2.8(1 + *a*_*M*_/*c*_4_)*μ*m. The initial cell cycle length decreases with the age of its mother and *g*_0_ in Eq (6) is modified to be *g*_0_/(1 + *a*_*M*_/*c*_5_). (iii) The frequency of successful matings of haploid cells is influenced by the ages of their mother cells, so that Eq (7) is modified to

$$P_{m}\left(a,a_{M}\right)=0.75-(\frac{a}{k_{3}}+\frac{a_{M}}{c_{6}}).$$

Here *c*_1_, *c*_2_, …, *c*_6_ are scaling parameters.

**Fig 7:**
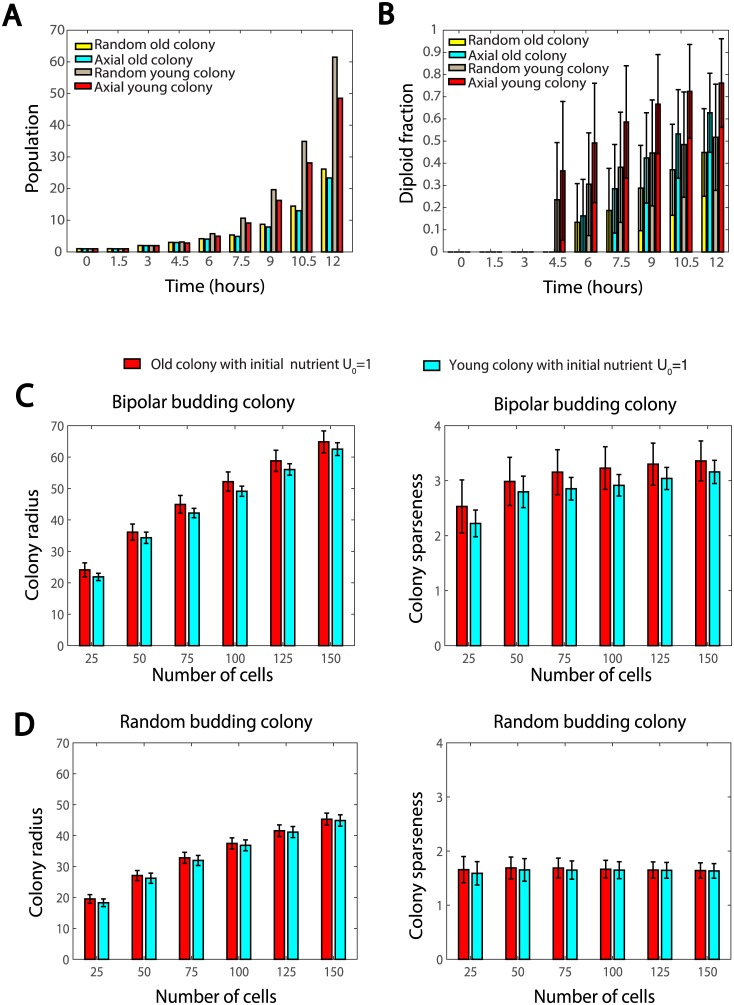
(A-B) Comparisons of mating features between old and young colonies formed by axial/random budding cells with mating type switch frequency 70%. (A) Time evolution of total populations of the colonies; (B) Diploid cell percentages of the colonies. (C-D) Comparison of colony radius and sparseness between old and young colonies with different budding patterns under limited nutrient *U*_0_ = 1. (C) Bipolar budding colonies. (D) Random budding colonies.

First, we tested how aging affects yeast colony by comparing two colonies: colonies initiated by a single haploid cell of age 0 (referred to as young colonies), and colonies initiated by a single haploid cell of age 30 (referred to as old colonies). Within each of these two colonies, both axial budding and random budding patterns are considered. It can be seen in Fig 7A that young colonies always have significantly larger populations than old colonies regardless of the budding patterns of the haploid cells. This result is expected, as the death probability increases with replicative age and the offsprings of old mothers need a few generations to fully rejuvenate. Fig 7B shows that mating occurs later in old colonies compared to young colonies: at 4.5 hours, the diploid cell percentage of old colonies is almost zero, while that of young colonies is over 20% on average. Fig 7B also shows that the old colonies tend to have a lower percentage of diploid cells, indicating lower mating efficiency in old colonies. Interestingly, although young colonies generally have higher mating efficiency, axial budding in haploid cells still shows its advantage: as time approaches 10 hours, the diploid cell percentage in an old colony with axial budding haploid cells (cyan bars in Fig 7B) exceeds that in a young colony with random budding haploid cells (gray bars in Fig 7B).

Young and old colonies initiated by four diploid cells of age 0 and of age 30 were also studied under limited nutrient. The first observation is that old colonies need a longer time to reach a certain population size, since offsprings of old mothers need several generations to rejuvenate. Fig 7C shows that if diploid cells bud in a bipolar manner, old colonies exhibit slightly larger colony radius and sparseness on average than young colonies; however, greater variance and more outliers are observed in old colonies. This may be explained by that most of the cells in a colony are young cells, and colony expansion is due to cell divisions at the periphery, which is also occupied by young cells (old cells reside in the core of the colony). On the other hand, if diploid cells bud in a random manner, almost no difference was observed between young and old colonies (Fig 7D).

In summary, our results suggest that the age of the colony determines the mating efficiency and how quickly the cell population increases, but does not affect the overall spatial distribution of cells.

## Discussion

In this paper, a two-dimensional agent-based model was developed to study budding yeast colonies with cell-type specific biological processes. Our model considers processes such as budding, mating cell death, consumption of nutrient and mating type switch. We investigated the roles of budding patterns, mating type switch frequency and growth inhibition induced by nutrient depletion in yeast colony development. Our findings reveal that axial budding pattern enhances mating efficiency at an early stage of colony development, and bipolar budding pattern improves colony expansion under nutrient limitation. Our results also suggest that mating type switch frequency might control the tradeoff between efficient diploidization and inbreeding. The effect of cellular aging was also studied. Based on the simulations, colonies initiated by an aged haploid cell show declined mating probability in an early stage of colony development but later recover as the rejuvenated offsprings become the majority. It was also shown that colonies initiated by aged diploid cells do not show disadvantage in colony expansion due to the fact that young cells contribute the most to colony expansion.

Our model can be extended to take into account intracellular signaling pathways and cellular responses. For simplicity and due to the lack of sufficient information, our model focused on a set of conceptual agent-based rules based on statistical results of experimental observations. However, one may also include the Cdc42 pathway or the cell cycle pathway for each individual cell to achieve a more realistic model. Another possible direction to extend our model is to include more detailed morphological changes induced by mating pheromone or nutrient depletion. In the current model, mating is only allowed when two cells of opposite types have direct contact, while in reality, cells may be able to sense mating pheromone over a longer distance and make projection toward the mating partners [33]. A more realistic field of mating pheromone described by reaction-diffusion equations as in [43] could be included. Elongated cellular morphology due to nutrient depletion may also be considered. The incorporation of cell morphological change may need to shift the current simple computational approach to a more involved numerical method, such as the level set or phase field methods [44], and the computational cost will increase drastically. Therefore, a more feasible first step would be using the current framework but making the directional growth possible as in [45], without considering the detailed cell shape change.

One major limitation of the agent-based approach is that the cell population size is restricted to a relatively small scale due to a high computational cost. For a relatively large population, it is often beneficial to consider on-lattice agent modeling or continuous model governed by continuum equations [46, 47]. However, these modeling approaches cannot capture some important biological phenomena at the small population scale, as studied in this paper.

In conclusion, our model is simple, but captures many essential characteristics of yeast colony development and our statistical results show good agreement with previous experiments and have verified some existing hypotheses. It can be extended to further understand the development of yeast colonies. It is worth noting that the model proposed here can serve as a framework to study multicellular organisms, especially systems such as tissues with stem cell lineage [48–50].

## Supporting information

S1 Fig Model parameters. (A) Estimated survival fractions using Eq (2). The blue curve for haploid cells is estimated through Fig. 1 in [20]. Since there is no available data for diploid cells, we simply estimated it to be the red curve based on the fact that diploid cells are longer-lived than haploid cells. (B) Estimated random budding probability using Eq (3). The curve for haploid cells is estimated through Fig. 3 in [19]. Due to lack of data, we use the same curve for diploid cells. (C) Estimated cell size using Eqs (4) and (5) and the data in [30, 31]. (D) Estimated cell cycle length using Eq (6) and the data in [30, 31].
(EPS)

S2 Fig A sample colony generated by a single haploid cell have a mixed types of cells. The simulation was run until 10.5 hours.
(EPS)

S3 Fig Demonstration of minimal covering circles. Minimal covering circles for diploid colonies. (A) A sample colony of bipolar budding diploid cells. (B) A sample colony of random budding diploid cells.
(EPS)

S4 Fig Samples of bipolar and random budding colonies with 150 cells under rich (*U*_0_ = 2) and poor (*U*_0_ = 1) nutrient conditions. (EPS)

S1 Text Numerical scheme solving the evolution of the nutrient field. (PDF)
