# Supplementary material for: A modeling study of budding yeast colony formation and its relationship to budding pattern and aging
Source: PLoS Comput Biol. 2017 Nov 9;13(11):e1005843. doi: 10.1371/journal.pcbi.1005843 (PMC5697893; doi:10.1371/journal.pcbi.1005843)
Supplement: S1 Text — (PDF) [file pcbi.1005843.s005.pdf]

S1 Text. Numerical scheme solving the evolution of the nutrient field

The nutrient field is defined across a square mesh of points. During each time step, nutrient on each grid point is consumed by nearby cells and diffuses over the domain. The method of lines is applied for the discretization of the nutrient equation (8). Second order central difference is used for approximating the Laplace operator and the forward Euler method is applied for solving the temporal evolution of the system. The discretized nutrient equation is

$$\begin{aligned}\frac{u_{i,j}^{n+1}-u_{i,j}^{n}}{\Delta t} =& D\left(\frac{u_{i+1,j}^{n}-2u_{i,j}^{n}+u_{i-1,j}^{n}}{\Delta x^2}+\frac{u_{i,j+1}^{n}-2u_{i,j}^{n}+u_{i,j-1}^{n}}{\Delta y^2}\right) \\ &- c_r\sum_{k=1}^{N(t)} e^{-\frac{\sqrt{(a_k-x_i)^2+(b_k-y_j)^2}}{c_d}}u_{i,j}^{n},\end{aligned} \tag{S1}$$

where $(a_k, b_k) = \vec{x}_k$ is the location of the k-th cell. The initial condition is $u(x, y, 0) = U_0$. No-flux conditions $\frac{\partial u}{\partial x} = 0, \frac{\partial u}{\partial y} = 0$ are imposed on the boundary of the domain. In the simulations, the time step Δt is chosen to be sufficiently small, compared to the cell cycle length of yeast agents; for the spatial mesh Δx and Δy are chosen to be sufficiently small, compared to the cell size of yeast agents. Our choices of $\Delta t, \Delta x$ and Δy satisfy stability conditions and also have high accuracy for convergence. We also checked that the conclusions in the main content do not depend on the mesh refinement. Our calculations are carried out by MATLAB.
